# Severe Methemoglobinemia Following Alkyl Nitrite Ingestion: A Case Report

**DOI:** 10.7759/cureus.89921

**Published:** 2025-08-12

**Authors:** Zane Kalik, Rabiu Momoh

**Affiliations:** 1 Internal Medicine, Medway Maritime Hospital, Kent, GBR; 2 Critical Care, Medway Maritime Hospital, Kent, GBR

**Keywords:** acquired methaemoglobinemia, alkyl nitrite, co-oximetry, emergencies, haemoglobin, methaemoglobinemia, overdose, saturations, substance abuse, toxicology

## Abstract

We report a case of a 40-year-old male who presented to the emergency department with central cyanosis, altered mental status, and hypoxia that was refractory to supplemental oxygen following ingestion of an alkyl nitrite product called "Liquid Gold". Arterial blood gas and co-oximetry confirmed a diagnosis of severe methemoglobinemia with a methemoglobin (MetHb) level in excess of 30%. The patient was treated with intravenous methylene blue and responded quickly both clinically and biochemically. This case further highlights the need to consider the dangers of recreational nitrite toxicity, the pitfalls of standard pulse oximetry in the setting of dyshemoglobinemia, and the value of prompt antidote therapy when faced with toxicological cases.

## Introduction

Amyl nitrite is a yellow, volatile liquid with a fruity odor that is commonly available for purchase and used recreationally for its aphrodisiac effects. This substance acts as a vasodilator and was once prescribed for treating angina. In some regions, it is also used as an antidote for cyanide poisoning. Slang terms associated with amyl nitrite include "ames," "aimies," "amyl," "amys," "bananas," "poppers," "hardcore," "pearl," "red rush," and "zap" [1]. Toxic effects can occur through absorption via the lungs, gastrointestinal tract, skin, and mucous membranes. Alkyl nitrites function as vasodilators and can oxidize ferrous iron (Fe^2+^) to ferric iron (Fe^3+^) in haemoglobin, leading to the formation of methemoglobin, which decreases the oxygen-carrying capacity of red blood cells. Toxic symptoms may develop within seconds after inhalation and are typically moderate and short-lived. After oral ingestion, symptoms can manifest within an hour and may persist for up to 12 hours. Toxidrome in this substance abuse will include the effects of severe vasodilation and, in more severe instances, methemoglobinemia, which can present with noticeable cyanosis, hypoxia, and central nervous system depression, among other issues [1].

## Case presentation

A 40-year-old male was brought in by paramedics to the hospital after he had been found with altered sensorium, grey-coloured skin, and vomiting at a sauna. Through his dizziness spells, he had reported that he had recreationally ingested content from a ‘Liquid Gold’ odorizer bottle that was given to him by another individual at a sauna. He had an ongoing intravenous plasmalyte infusion administered by the paramedic crew who attended to him in the community, and was on non-rebreathing face mask oxygen delivery with a reservoir bag at 15 litres/min flow. His past medical history included a diagnosis of atypical paranoid schizophrenia, and he was on treatment with clozapine. He was also a chronic smoker. Other past medical history included chronic constipation (requiring lactulose as needed), vitamin D deficiency, opioid dependence, and tremor.

**Figure 1:**
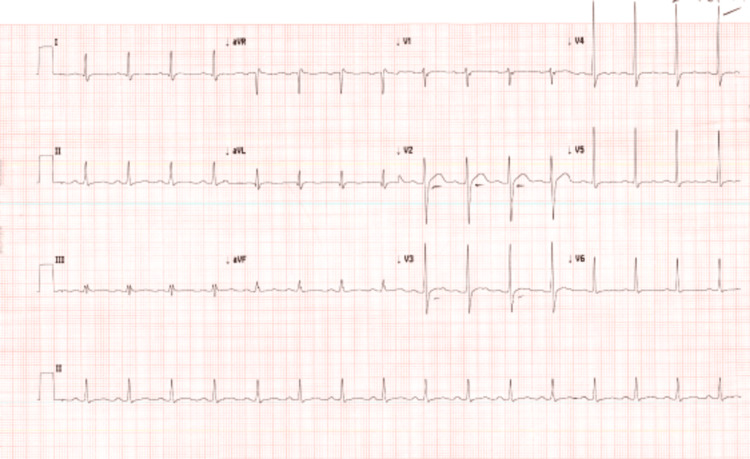
12-lead electrocardiogram study on the patient The graph shows an otherwise normal sinus rhythm with tachycardia and mild ST-segment change in the precordial lead V2.

The patient was both centrally and peripherally cyanotic, drowsy but arousable, and shivering. His initial oxygen saturation on the pulse oximeter was 77% even though he was being given 15 L/min of oxygen via a non-rebreather mask. Blood pressure was 118/60 mmHg, pulse 113 bpm (sinus rhythm), and temperature 36.5°C. Capillary refill time was 3 seconds. There was no trauma or signs of other toxidromes upon examination. Focal neurological deficits were absent. He was clammy and in rigors. His trachea was central, with an atraumatic chest with equal expansion and with normal tactile and vocal fremitus. Air entry was vesicular bilaterally and equal. He was, however, cyanotic. His heart rate was 113 beats per minute, sinus rhythm on a 12-lead electrocardiogram was done. His capillary refill time was 3 seconds. His mean arterial pressure was 70 mmHg. His abdomen was soft and non-tender. He was administered 15 L/min of oxygen delivery via a non-rebreathing face mask with a reservoir bag. A 12-lead electrocardiogram study done at admission showed an otherwise normal sinus rhythm with tachycardia but with a mild ST-segment elevation in precordial lead V2 (Figure 1).

Despite administration of oxygen at high flow, the patient's oxygen saturation was at its highest, 80-85%. A discrepancy between normal PaO₂ on arterial blood gas analysis and persistently low oxygen saturation on pulse oximetry led to a suspicion of dyshemoglobinemia. The blood that was sent for gas analysis was chocolate-brown in color. Co-oximetry revealed a methemoglobin level >30% (reference range: <1.5%), which confirmed an assessment of methemoglobinemia. The patient did not have a known history of glucose-6-phosphate dehydrogenase deficiency, a known disorder that may contraindicate the use of methylene blue therapy. The patient was promptly treated with 150 mg intravenous methylene blue (approximately 2 mg/kg) over five minutes. Cyanosis was resolved, and his mental status improved within 15-20 minutes, and oxygen saturations on pulse oximetry rose above 90%. He was admitted for monitoring. One hour after treatment, his MetHb was reduced to 14%; two hours after treatment, it fell to 0.8% (see Table 1). There were no further injections of methylene blue needed.

**Table 1:** Serial arterial blood gas and co-oximetry results PaO_2_: Partial pressure of oxygen, SpO_2_: Peripheral oxygen saturation.

Parameter	Initial	1 Hour Post-treatment	2 Hours Post-treatment	Reference Range
pH	7.33	7.34	7.41	7.35–7.45
PaO₂ (kPa)	14.9	31.9	8.4	10–13
Methemoglobin (%)	>30	14.0	0.8	<1.5
Lactate (mmol/L)	3.6	2.2	0.6	<2.0
SpO₂ (pulse oximetry)	83%	85%	98% (room air)	>95%

His other blood studies panel done on arrival to the hospital are shown in Table 2 below. He was monitored in the hospital for another 24 hours. He was thereafter reviewed by a psychiatry unit and was deemed safe for community discharge afterwards.

**Table 2:** Other relevant blood study results

Blood test	Result	Reference	Comment
Troponin T	5 ng/l	< 14ng/l	Normal study
C- Reactive Protein	1	< 5 mg/L	Normal study
Serum amylase	53	28 - 100 U/L	Normal study
Salicylate	< 15 mg/dl		Normal study
Paracetamol level	< 9	< 20 mcg/mL.	Normal study
Albumin	37 mg/l	35-50 g/l	Normal study
Total bilirubin	5 umol	< 17 umol/l	Normal study
Alkaline phosphatase	92 IU/l	30-130 IU/l	Normal study
Adjusted calcium	2.27 mmol/l	2.2-2.6 mmol/l	Normal study
Estimated glomerular filtration rate	73 ml/min	>90 ml/min	Normal study
sodium	140 mmol/l	135-145 mmol/l	Normal study
Serum potassium	3.8 mmol/l	3.5-5.5. mmol/l	Normal study
International normalized ratio	1	0.9-1.2	Normal study

## Discussion

This case illustrates a classic presentation of acute methemoglobinemia caused by alkyl nitrites, characterized by cyanosis that is unresponsive to oxygen therapy, alteration of mental status, and the diagnostic challenge posed by standard monitoring devices. Our patient's symptoms progressed rapidly to symptomatology after ingestion, showing characteristic signs of decreased pulse oximetry saturation in the presence of increased arterial oxygen tensions. The resultant methemoglobinemia was finally confirmed by co-oximetry. Increasing reporting of the public health danger posed by this drug of abuse is noted in the literature, including a similar case to ours published by O'Gorman et al. (2024), where amyl nitrite ingestion by a 39-year-old led to cyanosis and altered consciousness that was conservatively managed with just high flow oxygen therapy alone and a fall in measured methemoglobin level from 37.6% to 3% by the fourth hour [2]. In their case, methylene blue therapy was considered, but it was not used. There was a faster response to treatment with methylene blue therapy in our own case, with a fall from >30% methemoglobinemia to 0.8% after 2 hours [2]. The same route of administration (via oral ingestion) was noted in the case published by O'Gorman et al. and ours. This case also highlights the importance of early recognition and management of methemoglobinemia, which can occur in recreational drug abuse scenarios or from iatrogenic causes following administration of certain local anaesthetics (e.g., benzocaine, prilocaine) and antibiotics (e.g., dapsone, sulfamethoxazole, and sulfanilamide) [3].

Alkyl nitrites are strong oxidants that facilitate the transformation of iron in haemoglobin from its ferrous (Fe²⁺) to ferric (Fe³⁺) form, thereby causing the production of methemoglobin (MetHb) [4]. MetHb is unable to bind oxygen, thereby inhibiting the delivery of oxygen to tissues by decreasing the level of available hemoglobin as well as displacing the oxyhaemoglobin dissociation curve to the left [5]. In normal individuals, MetHb is kept at levels below 1-2% by NADH- and NADPH-dependent methemoglobin reductase mechanisms [6]. When these pathways are saturated with exogenous oxidizing substances, symptomatic methemoglobinemia results. Symptoms typically present at MetHb levels >10% (central cyanosis), with more ominous findings of confusion, dyspnea, and arrhythmias at levels >30% [7]. Concentrations above 50% may be life-threatening, with seizure, coma, or death [3]. Our patient had symptoms characteristic of MetHb >30%: altered mentation, cyanosis, and low pulse oximetry saturation that was unresponsive to oxygen. There is a higher potential for toxicity for ingestion of the drug of abuse under review over the inhalational route of administration due to slower metabolism and longer duration of action [8].

The differential diagnoses of severe methemoglobinemia would include conditions such as sulfhaemoglobinemia (as may occur with certain drugs (e.g., sulfonamides)). This has a similar presentation to methemoglobinemia, but sulfhemoglobin is irreversible and not treated with methylene blue. Cyanotic congenital heart disease may be accompanied by the presence of clubbing, auscultable cardiac murmur(s), and may be present as early as infancy. Patients with this condition respond poorly to oxygen, but echocardiography may reveal structural defects. Pulmonary embolism is another differential diagnosis that may be accompanied by sudden dyspnea, chest pain, and hypoxia. This usually responds to oxygen and is confirmed via imaging. Carbon monoxide poisoning is another differential diagnosis and may be accompanied by the presence of headache, confusion, and cherry-red skin. They have a normal PaO₂ but low oxygen delivery and are diagnosed via carboxyhemoglobin levels on a co-oximetry study. Other differential diagnoses include hemoglobin M disease and argyria (silver exposure) [3]. Our patient's case history, which was significant for an early confirmation of the use of the "Liquid Gold" product that contains amyl nitrate, provided an early opportunity to consider methemoglobinemia as the reason for his emergency presentation. He responded clinically well to methylene blue administration. His clinical examination did not reveal any auscultable cardiac murmur, and his co-oximetry study did not reveal the presence of carboxyhemoglobinemia. 

The primary treatment for patients showing symptoms or with moderate to severe methemoglobinemia (typically when levels exceed 20-30%) is intravenous methylene blue at a dose of 1-2 mg/kg, administered over five minutes [6]. Methylene blue serves as a synthetic electron carrier, promoting the reduction of MetHb through the NADPH-dependent pathway [9]. In most cases, clinical improvement is observed within 30 to 60 minutes, as was seen in our patient. Before giving methylene blue, it is often essential to assess for glucose-6-phosphate dehydrogenase (G6PD) deficiency, since individuals with this deficiency may struggle to produce sufficient NADPH. This can lead to worsening of symptoms or hemolysis [10]. For G6PD-deficient patients, alternative treatments like high-dose ascorbic acid, hyperbaric oxygen, or exchange transfusion may be appropriate [11]. Although rare, methylene blue can cause side effects such as serotonin syndrome (especially in those on SSRIs), hemolysis, and a blue-green discoloration of urine or skin [6]. Supportive care during nitrite poisoning remains crucial. Oxygen should be continued, even though it does not directly lower MetHb levels. Ongoing management involves repeated co-oximetry measurements, cardiac monitoring, and tracking blood lactate to evaluate tissue perfusion [12].

Beyond systemic consequences, the long-term recreational use of alkyl nitrites has been linked to skin and eye complications. Reported dermatologic reactions include popper's dermatitis [13], allergic contact dermatitis [14], and even cases where airborne exposure led to dermatitis in bystanders [15]. Ophthalmic issues such as poppers maculopathy and other visual impairments resulting from retinal toxicity, particularly following isopropyl nitrite exposure, have also been documented [16-18]. Survey data indicate a significant proportion of users report visual symptoms [19].

Many users are unaware of the true risks, especially when these substances are ingested rather than inhaled. Despite changes in nitrite formulations and longstanding awareness, with reports dating back to Israelstam et al., misuse remains common [19]. There are also accounts of accidental or food-related ingestion resulting in methemoglobinemia [20]. Clinicians should be vigilant for alkyl nitrite toxicity, particularly in nightlife or LGBTQ+ environments. Effective harm reduction strategies should prioritise educating users about the dangers of ingestion and promoting awareness of unusual clinical presentations [21].

## Conclusions

The present case further underscores the necessity of having a heightened awareness of the dangers of illicit nitrite toxicity and the early recognition of methemoglobinemia detection in risk groups and in environments where recreational drug use can be rampant. Methemoglobinemia should be considered in cyanotic patients who are not responsive to high-flow oxygen. Pulse oximetry is unreliable in methemoglobinemia, often plateauing at 85%. Co-oximetry is the gold standard of diagnosis and allows quantification of the hemoglobin species. Methylene blue is a first-line therapy for moderately to severely intense methemoglobinemia but is contraindicated in G6PD deficiency. Recreational alkyl nitrate use, particularly by ingestion, constitutes a significant threat to toxic methemoglobinemia and may warrant public health response and education.
